# Indigenous Uses and Pharmacological Activity of Traditional Medicinal Plants in Mount Taibai, China

**DOI:** 10.1155/2017/8329817

**Published:** 2017-02-20

**Authors:** Na Chang, Ziwen Luo, Dengwu Li, Huiying Song

**Affiliations:** ^1^College of Landscape Architecture and Arts, Northwest A&F University, Yangling, Shaanxi 712100, China; ^2^College of Forestry, Northwest A&F University, Yangling, Shaanxi 712100, China

## Abstract

This study was carried out to investigate the indigenous use and pharmacological activity of traditional medicinal plants of Mount Taibai, China. Pharmacological data were collected by conducting informal interviews with local experienced doctors practicing traditional Chinese medicine and via open-ended questionnaires on villagers. We conclude that the residents of Mt. Taibai possess rich pharmacological knowledge. This study may help identify high-value traditional medicinal plant species, promote economic development associated with local medicinal plants, and increase awareness from government departments.

## 1. Introduction

Ethnobotany is a plant science that studies historical and current uses of medicinal plants [1, 2]. It is of great significance for the conservation of ancient medicinal cultures, as well as for understanding changes in history and culture. It is also important for the conservation of traditional medicinal plant resources [3]. Furthermore, local residents with limited access to medical technology and equipment may benefit from traditional remedies, which can form an effective indigenous healthcare system. Such research may be significant in revealing important traditional medicinal plant species, often leading to the discovery of new drugs, and contributing to the local economy. Currently, millions of people in the developing world rely on traditional medicinal plants for primary healthcare, skin care, economic benefits, and cultural development. In areas where medical facilities are underdeveloped, traditional medicinal plants are especially important. Here, local residents may not distinguish between food, healthcare, and economic activity. This indigenous pharmacological knowledge of traditional medicinal plants should not be ignored. The global herbal remedies market was worth 19.4 billion USD in 1999 (not including shrubs and trees) [4]. Moreover, demand for traditional medicinal plants is increasing; for example, in India alone, the market is expanding at an annual rate of 20% [2]. The development of ethnobotany is expected to bring significant economic benefits, and scientific research is required to provide an evidence base for the development of the active ingredients of traditional medicines. Ethnobotany may also protect cultural heritage, inspire more studies of traditional medicines, and provide a basis for the discovery of new drugs.

Qinling is the most important northsouth geographical divide in China. The peak of Qinling is Mt. Taibai, which is the most important boundary in eastern mainland China in terms of climatic variation and the distribution of vegetation, and is especially significant because of its eastwest alignment and abundance of species [5]. Mt. Taibai has 1,850 species of plants belonging to 126 families, 25 of which are used in traditional Chinese medicine.

In recent years, interest has grown in the traditional medicinal plants of Mt. Taibai, both domestically and internationally. Attention has focused on biodiversity and pharmacological properties of individual species; however, few studies have attempted to evaluate their medicinal efficacy, or to explore the scientific basis of these plant medicines [6–8]. Although many of these plants have previously been investigated, most studies have been inconclusive. To provide a good evidence base for the usage of traditional medicinal plants, further studies should be carried out to investigate the distribution and usage of medicinal plants and critically evaluate their efficacy.

We investigated and documented traditional medicinal plants in Mt. Taibai and analyzed the treatment diversity of medicinal methods, identifying numerous plant parts, remedy formulations, and ailments that they were used to treat. We also evaluated the efficacy of these medicinal plants by comparing local usage with findings from published phytochemical and pharmacological studies. We believe that this research will not only help to stimulate the local economy, but also help to promote the protection and utilization of traditional medicinal plants.

## 2. Study Area and Methods

### 2.1. Study Area

**Figure 1:**
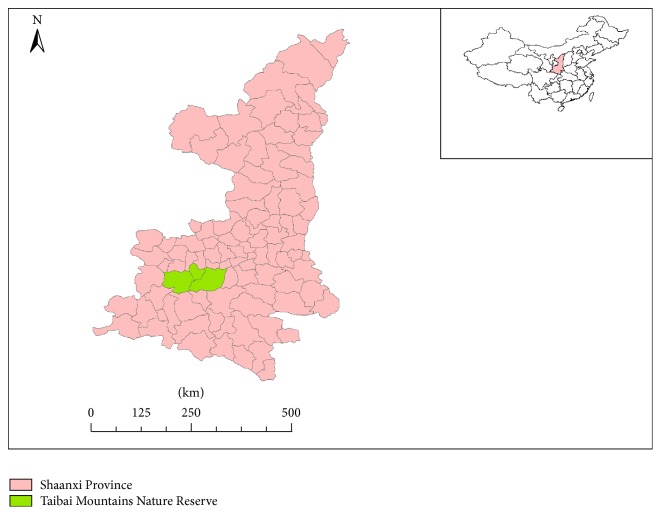
Location of Taibai Mountains Nature Reserve in Shaanxi, China.

Mt. Taibai (107°22′–107°51′ E, 33°49′–34°05′ N) is located in the center of the Qinling mountain range in Shaanxi Province, China. It covers Taibai County, the southern part of Mei County, and the southwestern part of Zhouzhi County. The elevation of the study region extends from 819 to 3767 m (see Figure 1) [9]. The Mt. Taibai Nature Reserve was designated in September 1965 by the government of Shaanxi Province. It contains a diverse range of flora, including 1,783 seed plant species (597 genera and 126 families), 325 bryophyte species (142 genera and 62 families), and 110 fern species (40 genera and 21 families), constituting approximately 60% of the flora of the Qinling range [10–12]. Interviews and surveys, as well as specimen collection, were undertaken in the Taibai Mountains (Mt. Taibai) Nature Reserve and the surrounding areas, including villages in Taibai County, Mei County, and Zhouzhi County.

### 2.2. Study Methods

Pharmacological data were collected by conducting interviews with local experienced doctors of traditional Chinese medicine, and open-ended questionnaires were given to villagers. Participants were selected to include plant collectors, plant cultivators, and plant traders. In total, nine experienced doctors of traditional Chinese medicine were interviewed; all were male, five were aged more than 50 years, and four were aged 40–50 years. In addition, 41 villagers (74% male, 26% female) participated in our open-ended questionnaire, 54% of whom were aged more than 40 years. The majority (54%) had no formal education, and 46% had primary school education, of whom 21% also had secondary school education. We gathered information on the altitudes of plant distributions, plant uses, the parts of plants that are used, their modes of utilization, the formulations of remedies, and the ailments that were treated using them. For species that could not be identified with certainty, specimens were collected for identification using references and further expert knowledge. The informant consensus factor *F*_IC_ was used to describe the variability of traditional medicinal plants. A high value of *F*_IC_ indicates good agreement on a particular ailment, whereas a low value of *F*_IC_ corresponds to poor agreement. High values of *F*_IC_ thus indicate particularly interesting species in the search for bioactive compounds. *F*_IC_ was calculated as follows [13]:

(1) $$\begin{array}{c} F_{IC}=\frac{\left(N_{ur}-N_{t}\right)}{\left(N_{ur}-1\right)}, \end{array}$$

where *N*_ur_ is the number of individual reports of plant use for a particular illness category and *N*_*t*_ is the total number of species used for this illness category.

To determine the variability, the reasonability of preparation methods, and the efficacy of the medicinal plants, we analyzed the altitudes at which the plants were grown, the taxonomic category, the parts of the plants that were used, the ailments that were treated, the chemical composition, and the pharmacological activity. Data were plotted using Sigmaplot 12.0, MapGIS 6.7, and Photoshop 6.0 for Windows.

## 3. Results and Discussion

### 3.1. Distribution at Different Altitudes

**Figure 2:**
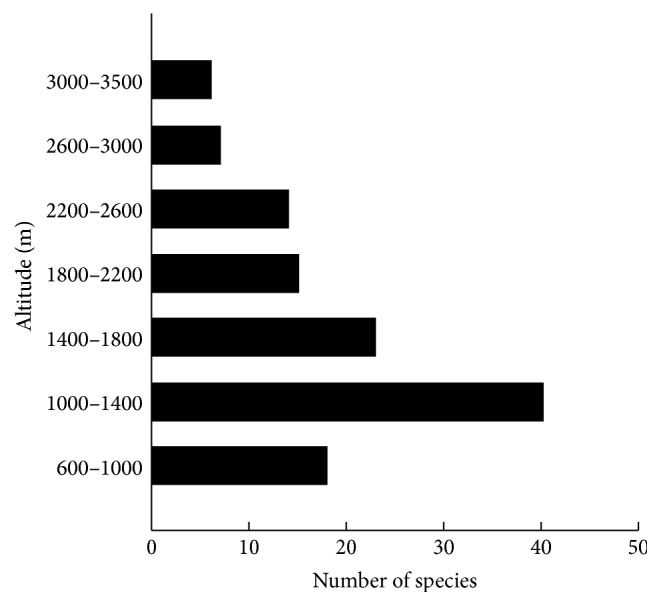
Distribution frequencies (number of species) of medicine plants.

We recorded a total of 50 species of traditional medicinal plants grown at various altitudes on Mt. Taibai. Forty species were found at altitudes of 1000–1400 m, 23 species at altitudes of 1400–1800 m, 18 species at altitudes of 600–1000 m, 15 species at altitudes of 1800–2200 m, 14 species at altitudes of 2200–2600 m, 7 species at altitudes of 2600–3000 m, and 6 species at altitudes of 3000–3500 m (Figure 2). Therefore, we concluded that altitudes in the range of 1000–1400 m represented the best sampling location.

### 3.2. Taxonomic Categories

**Figure 3:**
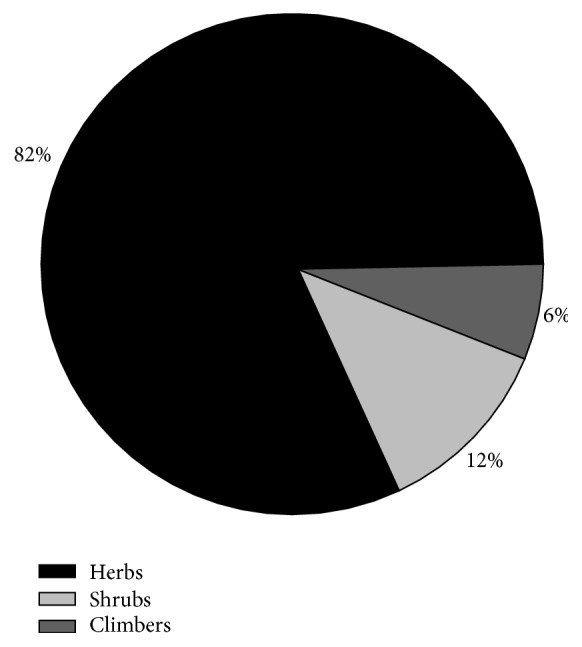
Percentage distributions of medicinal plant species according to life form.

All of these species were angiosperms, with 46 genera belonging to 32 families. There were 41 species of herb (82%), 6 species of shrub (12%), and 3 species of climber (6%; see Figure 3). Shrubs and grassland are important habitats for medicinal plants [14]. The herb layer is more complex and variable than the shrub layer, and the interaction between species is strong. Owing to the heterogeneity of the herb layer, it has rich species diversity [15]. It is believed that the more abundant the plant, the more the medicinal virtues it may possess [16, 17].

### 3.3. Part of the Plant Used for Medicine

**Figure 4:**
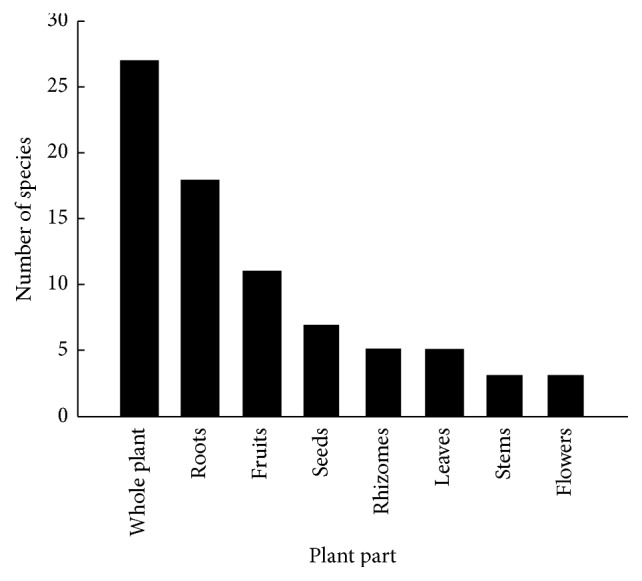
Use frequencies (number of species) of different plant parts in traditional medicine preparation.

Medicinal formulations can be prepared from roots, rhizomes, seeds, leaves, flowers, fruits, stems, or the whole plant. In this study, the most commonly used part was the whole plant (27 species), followed by roots (18 species), fruits (11 species), seeds (7 species), leaves (5 species), rhizomes (5 species), stems (3 species), and flowers (3 species) (Figure 4). The use of multiple plant parts was also recorded in some cases, including* Iris lactea*, where the leaves, roots, seeds, or flowers may be used for medicine, and* Acorus calamus*, where the roots, flowers, or leaves are used. It has been shown that some traditional medicinal plants may have effects when used in the form of preparations made using roots, leaves, and flowers. Deng and Hou [18] carried out chemical analyses and pharmacological experiments using 18 plant roots, leaves, and flowers, with clinical observations and a comparative study showing that many nonmedicinal parts of plants had medicinal value, which is significant for the development of new medicinal resources. Many traditional Chinese medicines in nonmedicinal parts of in-depth research have new insights, in the development of new resources.

### 3.4. Ailments Treated

**Figure 5:**
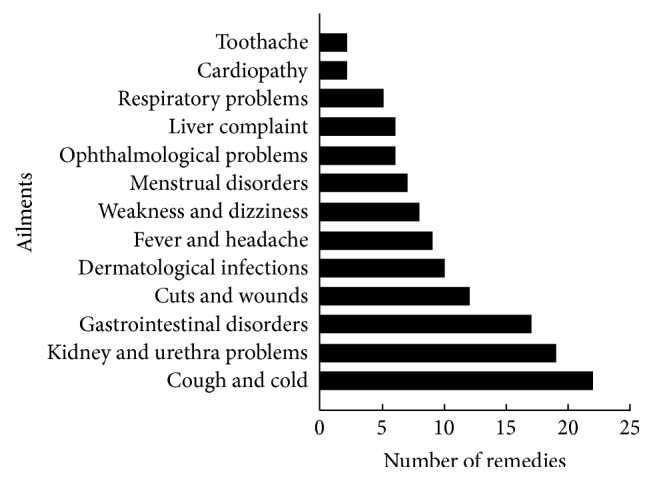
Number of remedies used for various ailments.

Gastrointestinal disorders, coughs, colds, urological problems, dermatological infections, heart diseases, fever, headaches, liver complaints, weakness, dizziness, respiratory problems, ophthalmological problems, cuts, and wounds were treated using traditional medicinal plants. Coughs and colds were treated with the greatest diversity of plant species (22 species), followed by urological problems (19 species) and gastrointestinal disorders (17 species). Respiratory problems, heart disease, and toothache were treated with the lowest diversity of plant species (5, 2, and 2, resp.) (Figure 5). Many species were used to treat multiple ailments, such as* Solanum nigrum*,* Origanum vulgare*,* Lespedeza bicolor*,* Lespedeza cuneata*,* Carum carvi,* and* Valeriana officinalis*, which were used to treat four to five ailments (Table 1). This suggests that there is significant potential value among these species. Some species were used to treat few ailments, such as* Acorus calamus*, which was used to treat only cough and toothache. Some species were used to treat only a single ailment, such as* Thlaspi arvense*, which was used to treat urological problems only (Table 1). The reasons why coughs, urological problems, and gastrointestinal disorders were treated with such a diversity of species may be related to the local climate conditions, living environment, and habits.

### 3.5. Formulations

**Figure 6:**
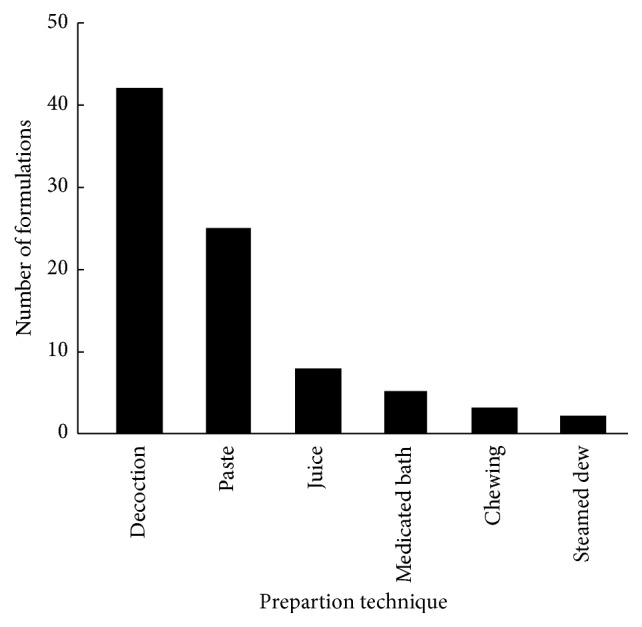
Use frequencies (number of medicinal formulations) of different remedy preparation techniques.

We found that 85 medicinal formulations were prepared using the 50 traditional medicinal plants identified in this study. Methods/applications included decoctions, pastes, juices, chewing, steaming, and medicated baths (Table 2). The most common formulation was decoction (42), followed by paste (25), juice (8), medicated bath (5), chewing (3), and steaming (2).* Polygonum aviculare*,* Portulaca oleracea*,* Sanguisorba officinalis*,* Lespedeza cuneata*,* Tribulus terrestris*,* Pyrola rotundifolia*,* Verbena officinalis*, and* Veronicastrum sibiricum* were processed into three different formulations (Figure 6). A total of 19 species were prepared as two different formulations, and all of the remaining 23 species were only prepared as a single formulation. Decoction was the most widely used preparation, which may be because of its simplicity and convenience (the processes of applying medicated baths, chewing, and steaming are more complicated or less convenient). Additional preparations have also been reported; for example, Chen et al. [19] described a preparation made using a combination of egg, tea leaves, and yellow wine, which was used as an embrocation to treat skin diseases, as well as the use of rice or other foods to enable swallowing or topical applications.

### 3.6. Informant Consensus Factor

The level of informant agreement was medium–high (mean *F*_IC_ = 0.65). *F*_IC_ values for most diseases were in the range of 0.60–0.70. Respiratory problems, menstrual disorders, and urological problems exhibited relatively low levels of consensus (*F*_IC_ = 0.56, 0.67, and 0.57, resp.). High values of *F*_IC_ were obtained for toothache and heart disease (0.88 and 0.83, resp.), showing that locals had reached good agreement on the plant species (*Polygonatum odoratum*,* Valeriana officinalis*,* Acorus calamus*, and* Asarum sieboldii*) to be used for these ailments. With the development of national medicine, a variety of herbs may contribute to traditional medicines and can complement the development of traditional Chinese medicine theory and practice [20]. These species may have significant value, so further investigation of their active compounds is warranted (Table 3).

### 3.7. Efficacy of Traditional Medicinal Plants

By collecting phytochemical and pharmacological data on the 50 traditional medicinal plants based on questionnaire data from local residents, and comparing this with bioefficacy data from literature reports, we found that the use of traditional medicinal plants in Mt. Taibai was consistent with known phytochemical or pharmacological properties in 84% of cases. In total, 28 medicinal species showed complete correspondence and 14 (*Polygonum viviparum*,* Rumex acetosa*,* Tribulus terrestris*,* Paeonia obovata*,* Thlaspi arvense*,* Polygonatum odoratum*,* Actinidia arguta*,* Astragalus chrysopterus*,* Lespedeza bicolor*,* Solanum nigrum*,* Cynanchum wilfordii*,* Metaplexis japonica*,* Daucus carota*, and* Anaphalis sinica*) showed partial correspondence. These results showing only partial phytochemical and pharmacological correspondence warrant further research into the uses of these plants. It was difficult to evaluate the pharmacological activity of the following eight species:* Iris lactea*,* Lespedeza cuneata*,* Oxalis acetosella*,* Sophora flavescens*,* Lepidium apetalum*,* Leonurus pseudomacranthus*,* Ranunculus sceleratus*, and* Cephalanoplos segetum*. Although many of these have been shown to contain active substances, further research is required to investigate their efficacy. The species* Oxalis acetosella*,* Sophora flavescens*, and* Leonurus pseudomacranthus* (Table 4) have not been widely studied, and their pharmacological activity is largely unknown. To help promote local economic development, we conclude that the previous 42 medicinal species should be given sufficient attention, but that the final eight species are also worthy of further research, with potential applications in drug development.

### 3.8. Suggestions for Further Exploitation and Protection

By comparing information on the use of traditional medicinal plants with the Chinese Materia Medica and other related research, we found that the properties of many of the identified species exhibited similar results. This shows that the use of traditional medicinal plants is self-consistent. This self-consistent knowledge of traditional medicinal plant species is extremely valuable and may promote research into the culture of traditional remedies and expedite the development of medicine. Therefore, knowledge of traditional medicinal plants is significant and should be conserved. Further development and utilization and effective cultivation and preparation methods, as well as education and regulation, are important strategies that can help exploit the benefits of these medicinal plants. Against this background, we make the following proposals.

First, regulations should be issued by government to control and standardize the use and trade of traditional medicinal plants. Second, the traditional knowledge of the use of medicinal plant is very important, but local residents may not take this seriously unless they realize the value of these traditional medicinal plants. Therefore, effective communication/publicity is important. Third, improvements should be made to the market for medical plants. Trade is an important factor in the use of medicinal plants, and protection for traders is therefore important. To promote economic development of local medicinal plants, protection and production should be taken into consideration. If such regulation is impractical, medicinal botanical gardens may be a good option. Training and demonstrations are important not only to preserve the germplasm resources of wild medicinal plants, but also to improve publicity. Finally, support from government is an important factor, as the implementation of the above strategies requires support from government. Only government has the capabilities to ensure sustainable development of these medicinal plant resources.

## 4. Conclusions

Mt. Taibai is rich in medicinal plant resources, and the local people possess a systematic and self-consistent knowledge of these native medicinal plants, including identification, application, and treatment modalities. Altitudes in the range of 1000–1400 m were found to be most promising for sample collection. Herbs were the most widely used plant species because of their abundance and the relative ease of collection, preparation, and storage. The most frequently used parts of plants were roots. Coughs and colds were the ailments that were treated with the greatest diversity of medicinal plant species. Decoction was the most widely used formulation.

The level of informant agreement was medium–high (mean *F*_IC_ = 0.65). The highest values of *F*_IC_ were obtained for toothache and heart disease (0.88 and 0.83, resp.), indicating good agreement in terms of the plant species used to treat these conditions (*P. odoratum*,* V. officinalis*,* A. calamus*, and* A. sieboldii*). These species therefore have significant value, and further research into their active compounds is warranted. We found 84% self-consistency of traditional plant use, which, together with scientifically proven phytochemical and pharmacological properties, indicates that traditional medical theories and formulations may be important and effective aspects of healthcare. We found that 14 species (*P. viviparum*,* R. acetosa*,* T. terrestris*,* P. obovata*,* T. arvense*,* P. odoratum*,* A. arguta*,* A. chrysopterus*,* L. bicolor*,* S. nigrum*,* C. wilfordii*,* M. japonica*,* D. carota*, and* A. sinica*) exhibited only partial correspondence in terms of pharmacological activity and that 8 (*I. lacteal*,* L. cuneata*,* O. acetosella*,* S. flavescens*,* L. apetalum*,* L. pseudomacranthus*,* R. sceleratus*, and* C. segetum*) showed inconclusive results; however, there may be significant potential for the use of these plants, and further investigation is warranted for all species. The chemical compounds contained in the species* O. acetosella*,* S. flavescens*, and* L. pseudomacranthus* have not been reported, and their pharmacological activity is largely unknown. More complete and systematic knowledge of the phytochemical and pharmacological properties of traditional medicinal plants is desirable, and medicinal plants have considerable potential for healthcare applications. Therefore, we attach importance to the conservation of biodiversity, as well as traditional knowledge of the medicinal use of these plants. Proper management and exploitation of traditional medicinal plants may provide a sustainable source of income for local communities. This provides incentives for conservation to ensure the long-term availability of these traditional medicinal plants, both for use as indigenous drugs and for commercial exploitation.

## Figures and Tables

**Table 1:** Medicinal plants used to cure various ailments.

Ailment	Plants
Cough and cold	*Acorus calamus* Linn., *Polygonatum odoratum* Mill., *Iris lactea* Pall. var. chinensis Koidz., *Humulus scandens* (Lour.) Merr., *Asarum sieboldii* Miq., *Pseudostellaria heterophylla* (Miq.) Pax ex Pax et Hoffm., *Lepidium apetalum* Willd.,* Lespedeza bicolor* Turcz.,* Hippophae rhamnoides* Linn., *Daucus carota* Linn., *Carum carvi* Linn.,* Pyrola rotundifolia* Linn. subsp. Chinensis H. Andres., *Metaplexis japonica* (Thunb.) Makino., *Origanum vulgare* Linn., *Veronicastrum sibiricum* (Linn.) Penell., *Lonicera japonica* Thunb., *Valeriana officinalis* Linn., *Xanthium sibiricum* Patrin ex Widder., *Bidens parviflora* Willd., *Cephalanoplos segetum* (Bge.) Kitam., *Anaphalis sinica* Hance., *Arctium lappa* Linn.

Kidney and urethra problems	*Houttuynia cordata* Thunb., *Humulus scandens*, Polygonum aviculare Linn., *Portulaca oleracea* Linn., *Ranunculus sceleratus* Linn., *Thlaspi arvense* Linn., *Astragalus chrysopterus* Bge., *Lespedeza bicolor*, *Daucus carota*, *Carum carvi*, *Diospyros lotus* Linn., *Cynanchum wilfordii* (Maxim.) Hemsl., *Metaplexis japonica*, *Leonurus pseudomacranthus* Kitag., *Origanum vulgare*, *Plantago depressa* Widd., *Plantago asiatica* Linn., *Valeriana officinalis*, *Cephalanoplos segetum*

Gastrointestinal disorders	*Rumex acetosa* Linn., *Portulaca oleracea*, *Sophora flavescens* Ait., *Lespedeza cuneata* (Dum. Cours.) G. Don., *Geranium sibiricum* Linn., *Daphne giraldii* Nitsche., *Hippophae rhamnoides*, *Daucus carota*, *Carum carvi*, *Origanum vulgare*, *Solanum nigrum* Linn., *Plantago depressa*, *Lonicera japonica*, *Valeriana officinalis*, *Artemisia annua* Linn., *Bidens parviflora*, *Anaphalis sinica*

Cuts and wounds	*Gymnadenia conopsea* R. Br., *Polygonum viviparum* Linn., *Paeonia obovata* Maxim., *Lepidium apetalum*, *Sedum aizoon* Linn., *Sanguisorba officinalis* Linn., *Astragalus chrysopterus*, *Lespedeza cuneata*, *Oxalis acetosella* Linn., *Geranium sibiricum*, *Pyrola rotundifolia*, *Verbena officinalis* Linn.

Dermatological infections	*Rumex acetosa*, *Ranunculus japonicus* Thunb., *Sophora flavescens*, *Astragalus chrysopterus*, *Oxalis acetosella*, *Diospyros lotus*, *Verbena officinalis*, *Solanum nigrum*, *Artemisia annua*, *Bidens parviflora*

Fever and headache	*Humulus scandens*, *Polygonum viviparum*, *Portulaca oleracea*, *Lespedeza bicolor*, *Origanum vulgare*, *Solanum nigrum*, *Veronicastrum sibiricum* (Linn.) Penell, *Artemisia annua*, *Anaphalis sinica*

Weakness and dizziness	*Polygonatum odoratum*, *Gymnadenia conopsea*, *Pseudostellaria heterophylla*, *Lepidium apetalum*, *Lespedeza bicolor*, *Carum carvi*, *Cynanchum wilfordii*, *Metaplexis japonica*

Menstrual disorders	*Ranunculus sceleratus*, *Paeonia obovata*, *Actinidia arguta* (Sieb. et Zucc.) Planch., *Pyrola rotundifolia*, *Verbena officinalis*, *Leonurus pseudo-macranthus*, *Origanum vulgare*

Ophthalmological problems	*Lespedeza cuneata*, *Tribulus terrestris* Linn., *Diospyros lotus*, *Solanum nigrum*, *Plantago depressa*, *Plantago asiatica*

Liver complaint	*Iris lactea*., *Gymnadenia conopsea*, *Tribulus terrestris*, *Actinidia arguta*, *Plantago depressa*, *Plantago asiatica*

Respiratory problems	*Houttuynia cordata*, *Ranunculus sceleratus*, *Lespedeza cuneata*, *Diospyros lotus*, *Solanum nigrum*

Heart diseases	*Polygonatum odoratum*, *Valeriana officinalis*

Toothache	*Acorus calamus*, *Asarum sieboldii*

**Table 2:** Common forms of preparation methods for remedies made of medicinal plants.

Preparation method	Description
Paste	Fresh plant parts are crushed with a stone pestle and mortar.
Juice	Obtained by squeezing or crushing plant parts. Sometimes requires addition of other liquids for dilution.
Chewing	Fresh plant parts are chewed.
Steamed dew	Drugs are extracted from medicinal raw materials by distillation and then modulated into distilled liquid for drinking, wiping, or other uses.
Decoction	Plant parts are boiled in water and the extract (crude drug) is used.
Medicate bath	Fresh flowers or other plant parts are immersed in hot water for bathing.

**Table 3:** Informant consensus factor (*F*_IC_) for different ailment categories.

Ailment	Number of taxa (*N*_*t*_)	Number of use reports (*N*_ur_)	Informant consensus factor (*F*_IC_)
Cough and cold	22	54	0.60
Kidney and urethra problems	19	43	0.57
Gastrointestinal disorders	17	47	0.65
Cuts and wounds	14	41	0.68
Dermatological infections	10	25	0.63
Fever and headache	9	21	0.60
Weakness and dizziness	8	18	0.59
Menstrual disorders	7	15	0.57
Ophthalmological problems	6	14	0.62
Liver complaint	6	17	0.69
Respiratory problems	5	10	0.56
Heart diseases	2	7	0.83
Toothache	2	9	0.88
Total	127	321	

**Table 4:** Comparison of local use and phytochemical/pharmacological properties of medicinal plants.

Species	The main usage in local place (present study)	Phytochemical/pharmacological properties (literature review)	Local use coherent with known phytochemical/pharmacological properties
*Portulaca oleracea*	Whole plant soup is taken for enteritis and constipation	Alkaloid extract may possess anti-inflammatory properties [21]	Yes

*Iris lactea*	Seeds and flowers are applied on heat-clearing and detoxifying	Containing more than seven kinds of flavonoids [22], but seldom pharmacological research	Unknown

*Gymnadenia conopsea*	Whole plant and rhizomes are used for wounds, weakness, and dizziness	Antiallergic effect [23]	Yes

*Houttuynia cordata*	Whole plant is taken for respiratory and kidney problems	Anti-inflammatory and virucidal effects [24, 25]	Yes

*Humulus scandens*	Whole plant is used for fever, cough, and urethra problem	Antibacterial, antihypertensive, and antiphlogistic properties [26]	Yes

*Polygonum viviparum*	Rhizomes are taken for wounds, cough, and cold	Antioxidative activity [27, 28]	Partial

*Rumex acetosa*	Whole plant is applied on dermatological infections and gastrointestinal disorders	Antimutagenicity and antigenotoxic activity [29], but seldom pharmacological research	Partial

*Plantago depressa*	Used for hepatitis and seeds are applied on diarrhea or eye diseases	Hypoglycemia and lipids regulating effects [30]	Yes

*Polygonum aviculare*	Whole plant is used for kidney and urethra problems	Diuretic, antihypertensive, antibacterial, and antioxidant effect [31]	Yes

*Carum carvi*	Fruits are taken for dyspepsia, coughs, diuresis, and stomachache	Antioxidant, hepatoprotective, and diuretic properties [32, 33]	Yes

*Pseudostellaria heterophylla*	Roots are taken for cough, weakness, and dizziness	Antifungal and immunostimulating activities [34, 35]	Yes

*Xanthium sibiricum *	Leaves are applied on wind chill and colds	Bacteriostatic and antifungal activities [36, 37]	Yes

*Tribulus terrestris*	Fruits are applied for eye diseases, menstrual disorders, and liver problems	Having several effects on central neural system, sex function, and muscular system [38]	Partial

*Ranunculus japonicas*	Used for dermatological infections	Analgesic and anti-inflammatory effects [39]	Yes

*Paeonia obovate*	Roots are taken for cough and menstrual disorders	Hypoglycemic activity and immunocompetence of paeoniflorin [40]	Partial

*Thlaspi arvense*	Whole plant and seeds are applied for kidney and urethra problems	Antibacterial and antifungal activities [41]	Partial

*Sedum aizoon*	Whole plant an roots are taken for cuts and wounds	Improving the immune function and relieving swelling and pain [42]	Yes

*Plantago asiatica*	Used for diarrhea, hepatitis, and red swollen and painful eye	Antiviral and immunomodulatory effects [43]	Yes

*Sanguisorba officinalis*	Roots are used for cuts and wounds	Antimicrobial activity [44]	Yes

*Lonicera japonica*	Used for gastrointestinal disorders, colds, and fever	Anti-inflammatory activity [45]	Yes

*Valeriana officinalis*	Roots are taken for cough, heart diseases, and lubricating the intestines	Having effect on circulatory system and respiratory system [46]	Yes

*Polygonatum odoratum*	Roots are taken for palpitation, coughs, and physical weakness	Hypoglycemic effects [47]	Partial

*Acorus calamus*	Used for febrile pain, colds, and toothache	Reduction of body temperature and potentiation of hypnotic activity [48]	Yes

*Lespedeza cuneata*	Used for Gastrointestinal disorders, wounds, and respiratory problems	Contains tannins [49]	Unknown

*Oxalis acetosella*	Used for wounds and dermatological infections	Seldom report on physiological activity	Unknown

*Geranium sibiricum*	Whole plant and roots are taken for wounds and gastrointestinal disorders	Antibacterial and anti-inflammatory activities [50]	Yes

*Sophora flavescens*	Root are used for dermatological infections and gastrointestinal disorders	Contains matrine [51]	Unknown

*Actinidia argute*	Used for menstrual disorders and liver complaint	Contains sesquiterpenes, monoterpenes, benzene, and other compounds [52]	Partial

*Daphne giraldii*	Used for headache, arthralgia, and gastrointestinal disorders	Anti-inflammatory analgesic activity [53]	Yes

*Astragalus chrysopterus*	Whole plant is taken for wounds, heart diseases, and dermatological infections	Contains soyasaponin, triterpenoid, glycoside daucosterol, beta-sitosterol, and other compounds [54]	Partial

*Lespedeza bicolor*	Leaves and stems are applied for cough, fever, weakness, and kidney problems	Contains ethyl caffeate, caffeic acid, protocatechuic acid, betulinic acid, *β*-sitosterol, and many active compounds [55]	Partial

*Asarum sieboldii*	Roots are applied for cold, headache, and toothache	Antinociceptive effects [56]	Yes

*Hippophae rhamnoides*	Fruits are taken for coughs, colds, and gastrointestinal disorders	Antioxidant and immunomodulatory properties [57]	Yes

*Solanum nigrum*	Whole plant is taken for stomachache, headache, hot eyes, and faucitis	Gastric antiulcerogenic effects [58]	Partial

*Pyrola rotundifolia*	Used for cough, wounds, and menstrual disorders	Anti-inflammatory and analgesic activities [59]	Yes

*Origanum vulgare*	Used for colds, fever, vomiting, and menstrual disorder	Antimicrobial and cytotoxic activities [59]	Yes

*Lepidium apetalum*	Seeds are applied for fending off the cold and coughs and nourishing	Contains flavonoids [60]	Unknown

*Cynanchum wilfordii*	Roots are taken for weakness and kidney problems	Contains more than eight c21 steroidal glycosides [61]	Partial

*Metaplexis japonica*	Used for cough, dizziness, and urethra problems	Anticancer activity and improving immune function [62]	Partial

*Verbena officinalis*	Whole plant is used for wounds, dermatological infections, and menstrual disorders	Anti-inflammatory and analgesic activity [63]	Yes

*Leonurus pseudomacranthus *	Used for menstrual disorders and kidney and urethra problems	Seldom report on physiological activity	Unknown

*Veronicastrum sibiricum*	Whole plant and roots are taken for cough, fever, and headache	Anti-inflammatory and analgesic activities [64]	Yes

*Daucus carota*	Fruits are applied on invigorating stomach, coughs, nourishing, cystolith, and kidney stone	Hepatoprotective activity [65]	Partial

*Diospyros lotus*	Fruit juice is applied for malaria, diarrhea, and removal of black spots	Antioxidant and antiproliferative activity [66]	Yes

*Anaphalis sinica*	Whole plant is used for fever, cough, and gastrointestinal disorders	More than twenty components were isolated and many flavonoids were identified [67]	Partial

*Ranunculus sceleratus*	Used for phlegm, menstrual disorder, and diuresis	Many chemical compounds were detected [68], but seldom pharmacological research	Unknown

*Bidens parviflora*	Whole plant is used for cough, dermatological infections, and gastrointestinal disorders	Antihyperlipidemia, anti-inflammatory activities and protecting stomach [22]	Yes

*Cephalanoplos segetum*	Used for cough, kidney, and urethra problems	Contains high content of chlorogenic acid [69], but seldom pharmacological research	Unknown

*Artemisia annua*	Used for fever, intestinal tract disease, and skin disease	Antibacterial and antioxidant activities [70]	Yes

*Arctium lappa*	Fruits are taken for coughs, fever, and sore swollen throat	Anti-inflammatory activity [71]	Yes

## References

[1] Njoroge N. G., Bussmann W. R., Gemmill B. (2004). Utilization of weed species as source of traditional medicines in central Kenya. *Lyonia*.

[2] Pei S. (2008). Review on two decades development of ethnobotany in China. *Acta Botanica Yunnanica*.

[3] Gemedo-Dalle T., Maass B. L., Isselstein J. (2005). Plant biodiversity and ethnobotany of Borana pastoralists in southern Oromia, Ethiopia. *Economic Botany*.

[4] Pierce A. R., Laird S. A. (2003). In search of comprehensive standards for non-timber forest products in the botanicals trade. *International Forestry Review*.

[5] Tang Z., Fang J. (2006). Temperature variation along the northern and southern slopes of Mt. Taibai, China. *Agricultural and Forest Meteorology*.

[6] Zhang J., Du S., Duan X., Zhang S. (2007). Effects of ultrahigh pressure processing on the physicochemical characteristics of Taibai Kudzu starch. *Nongye Gongcheng Xuebao/Transactions of the Chinese Society of Agricultural Engineering*.

[7] Yue M., Zhou H. X. (1996). Diversity of higher plants in deciduous broadleaved forests on the northern slope of Taibai Mountain. *Acta Botanica Yunnanica*.

[8] Zhang L., Fang J. Y. (2004). Reserves and species diversity of soil seed banks in four types of forest on Mt. Taibai, Qinling Mountains. *Biodiversity Science*.

[9] Tang L., Li T., Li D., Meng X. (2014). Elevational patterns of plant richness in the Taibai Mountain, China. *Scientific World Journal*.

[10] Ren X., Yang G., Zhu F. (2012). Plant communities, species richness and life-forms along elevational gradients in Taibai Mountain, China. *African Journal of Agricultural Research*.

[11] Ying J. S., Li Y. F., Guo Q. F. (1990). Observations on the flora and vegetation of Taibai Shan, Qinling Mountain Range, Southern Shaanxi, China. *Acta Phytotaxonomica Sinica*.

[12] Kang Y., Łuczaj Ł., Kang J., Zhang S. (2013). Wild food plants and wild edible fungi in two valleys of the Qinling Mountains (Shaanxi, central China). *Journal of Ethnobiology and Ethnomedicine*.

[13] Uprety Y., Asselin H., Boon E. K., Yadav S., Shrestha K. K. (2010). Indigenous use and bio-efficacy of medicinal plants in the Rasuwa District, Central Nepal. *Journal of Ethnobiology and Ethnomedicine*.

[14] Wang W., Wu Y. N., Liu Y. (2015). Survey on the resources of medicinal angiosperm in Xunyang County. *Journal of Shaanxi College of Traditional Chinese Medicine*.

[15] Zang Y., Dong G. H., Du Y. L. (2013). Flora characteristics of wild medicinal plants in typical vegetation areas in Taibai Mountain. *Journal of Northwest A&F University*.

[16] Shrestha P. M., Dhillion S. S. (2003). Medicinal plant diversity and use in the highlands of Dolakha district, Nepal. *Journal of Ethnopharmacology*.

[17] Coe F. G., Anderson G. J. (1996). Ethnobotany of the garífuna of Eastern Nicaragua. *Economic Botany*.

[18] Deng J. G., Hou X. T. (2012). Overview of traditional Chinese medicinal parts. *Journal of Guangxi University of Chinese Medicine*.

[19] Chen G. X., Xu L., Luo W. (2011). Study on traditional medicinal plant resource usage of Tujia nationality trough ethnobotanical approaches in the Yongding District of the Zhangjiajie City. *Journal of MUC (Natural Sciences Edition)*.

[20] Yang B. D. (1994). Discussion on the choice of non medicinal parts of traditional Chinese Medicine. *Research on Traditional Chinese Medicine*.

[21] Chan K., Islam M. W., Kamil M. (2000). The analgesic and anti-inflammatory effects of *Portulaca oleracea* L. subsp. sativa (Haw.) Celak. *Journal of Ethnopharmacology*.

[22] Wang Q., Zhang Y. N., Chen F. H. (2009). Research development in chemical constituents and pharmacological action of total flavonoids of *Bidens bipinnata* L.. *Anhui Medical and Pharmaceutical Journal*.

[23] Matsuda H., Morikawa T., Xie H., Yoshikawa M. (2004). Antiallergic phenanthrenes and stilbenes from the tubers of *Gymnadenia conopsea*. *Planta Medica*.

[24] Lu H. M., Liang Y. Z., Yi L. Z., Wu X. J. (2006). Anti-inflammatory effect of Houttuynia cordata injection. *Journal of Ethnopharmacology*.

[25] Hayashi K., Kamiya M., Hayashi T. (1995). Virucidal effects of the steam distillate from Houttuynia cordata and its components on HSV-1, influenza virus, and HIV. *Planta Medica*.

[26] Gong L. X., Ding Z. P. (2009). Research advance of Humulus scandens and flavonoids. *Journal of Anhui Agricultural Sciences*.

[27] Li Z. D., Chen X. R., Li P. (2010). Identification of *Polygonum viviparum* endophytic bacteria Z5 and determination of the capacity to secrete IAA and antagonistic capacity towards pathogenic fungi. *Acta Prataculturae Sinica*.

[28] Zhou D. W., Zhu W. Y., Teng Z. H. (2003). Antioxidative compounds of *Polygonum viviparum* L. from different altitudes. *Chinese Journal of Applied & Environmental Biology*.

[29] Lee N.-J., Choi J.-H., Koo B.-S. (2005). Antimutagenicity and cytotoxicity of the constituents from the aerial parts of Rumex acetosa. *Biological and Pharmaceutical Bulletin*.

[30] Wu F.-H., Liang J.-Y., Yu P., Cai S.-F. (2005). Studies on the hypoglycemia and lipids regulating effects of *Plantago depressa* var. montata. *China Journal of Chinese Materia Medica*.

[31] Xu Y., Li M. M., Liu Z. H. R. (2012). Research progress in chemical constituents and pharmacological activities of *Polygonum aviculare* L.. *Journal of Anhui Agricultural University*.

[32] Samojlik I., Lakić N., Mimica-Dukić N., Daković-Švajcer K., Božin B. (2010). Antioxidant and hepatoprotective potential of essential oils of coriander (*Coriandrum sativum* L.) and Caraway (*Carum carvi* L.) (Apiaceae). *Journal of Agricultural and Food Chemistry*.

[33] Lahlou S., Tahraoui A., Israili Z., Lyoussi B. (2007). Diuretic activity of the aqueous extracts of *Carum carvi* and *Tanacetum vulgare* in normal rats. *Journal of Ethnopharmacology*.

[34] Wang H. X., Ng T. B. (2006). Concurrent isolation of a Kunitz-type trypsin inhibitor with antifungal activity and a novel lectin from *Pseudostellaria heterophylla* roots. *Biochemical and Biophysical Research Communications*.

[35] Wong C. K., Leung K. N., Fung K. P., Choy Y. M. (1994). The immunostimulating activities of anti-tumor Polysaccharides from *Pseudostellaria heterophylla*. *Immunopharmacology*.

[36] Fu M., Liu S. G., Wu X. J. (2005). Stuies on bacteriostastic comparison of *Rubus tephrodes* Hance and *Xanthium sibiricum* Patrin. *Huazhong Shifan Daxue Xuebao (Ziran Kexue Ban)*.

[37] Qi L. Y., Liu L. L., Yu P. R. (2008). Preliminary studies on antifungal activity of *Xanthium sibiricum* and the endophytic fungi. *Journal of Anhui Agricultural Sciences*.

[38] Chu S. D., Qu W. J., Li M., Cao Q. (2003). Research advance on chemical component and pharmacological action of tribulus terrestris. *Chinese Wild Plant Resources*.

[39] Cao B.-J., Meng Q.-Y., Ji N. (1992). Analgesic and anti-inflammatory effects of Ranunculus japonicus extract. *Planta Medica*.

[40] Hu N., Xu H. Y., Chen Z. W. (2007). The pharmacology research progress of *Paeoniflorin*. *Journal of Qiqihar University of Medicine*.

[41] Pedras M. S. C., Chumala P. B., Suchy M. (2003). Phytoalexins from Thlaspi arvense, a wild crucifer resistant to virulent *Leptosphaeria maculans*: structures, syntheses and antifungal activity. *Phytochemistry*.

[42] Meng F., Luo X., Gong J. O. (2010). verview of pharmacological research of *Sedum* L.. *Journal of Liaoning University of Traditional Chinese Medicine*.

[43] Chiang L.-C., Chiang W., Chang M.-Y., Lin C.-C. (2003). *In vitro* cytotoxic, antiviral and immunomodulatory effects of *Plantago major* and *Plantago asiatica*. *American Journal of Chinese Medicine*.

[44] Kokoska L., Polesny Z., Rada V., Nepovim A., Vanek T. (2002). Screening of some Siberian medicinal plants for antimicrobial activity. *Journal of Ethnopharmacology*.

[45] Lee S. J., Son K. H., Chang H. W., Kang S. S., Kim H. P. (1998). Antiinflammatory activity of *Lonicera japonica*. *Phytotherapy Research*.

[46] Yang Q., Ju A. H., Bai W. F. (2008). The research advance in chemical constituents and pharmacology activity for the *Valeriana officinalis* Linn. var. latiofolia Miq. *The Chinese Journal of Modern Applied Pharmacy*.

[47] Chen H., Feng R., Guo Y., Sun L., Jiang J. (2001). Hypoglycemic effects of aqueous extract of *Rhizoma Polygonati Odorati* in mice and rats. *Journal of Ethnopharmacology*.

[48] Dandiya P. C., Cullumbine H. (1959). Studies on *Acorus calamus* (III): some pharmacological actions of the volatile oil. *Journal of Pharmacology and Experimental Therapeutics*.

[49] Lange K. C., Olcott D. D., Miller J. E. (2006). Effect of sericea lespedeza (*Lespedeza cuneata*) fed as hay, on natural and experimental *Haemonchus contortus* infections in lambs. *Veterinary Parasitology*.

[50] Shim J.-U., Oh P.-S., Lim K.-T. (2009). Anti-inflammatory activity of ethanol extract from Geranium sibiricum Linne. *Journal of Ethnopharmacology*.

[51] Lai J.-P., He X.-W., Jiang Y., Chen F. (2003). Preparative separation and determination of matrine from the Chinese medicinal plant Sophora flavescens Ait by molecularly imprinted solid-phase extraction. *Analytical and Bioanalytical Chemistry*.

[52] Takano F., Tanaka T., Tsukamoto E., Yahagi N., Fushiya S. (2003). Isolation of (+)-catechin and (−)-epicatechin from *Actinidia arguta* as bone marrow cell proliferation promoting compounds. *Planta Medica*.

[53] Ruan Z., Chen R. J., Yu Y. Y. (2009). Research progresses on chemical constituents of Genus *Daphne genus* and their bioactivities. *Food Science*.

[54] Wang H. K., He K., Xu H. X. (1990). The structure of astrachrysosid A and the study of 2D-NMR on astrasieversianin XV and 7,2'-dihydroxy-3',4'-dimethoxy-isoflavane-7-O-beta-D-glycoside. *Acta Pharmaceutica Sinica*.

[55] Maximov O. B., Kulesh N. I., Stepanenko L. S., Dmitrenok P. S. (2004). New prenylated isoflavanones and other constituents of *Lespedeza bicolor*. *Fitoterapia*.

[56] Kim S.-J., Zhang C. G., Lim J. T. (2003). Mechanism of anti-nociceptive effects of *Asarum sieboldii* Miq. Radix: potential role of bradykinin, histamine and opioid receptor-mediated pathways. *Journal of Ethnopharmacology*.

[57] Geetha S., Sai Ram M., Singh V., Ilavazhagan G., Sawhney R. C. (2002). Anti-oxidant and immunomodulatory properties of seabuckthorn (*Hippophae rhamnoides*)—an in vitro study. *Journal of Ethnopharmacology*.

[58] Akhtar M. S., Munir M. (1989). Evaluation op the gastric antiulcerogenic effects of Solanum nigrum, *Brassica oleracea* and *Ocimum basilicum* in rats. *Journal of Ethnopharmacology*.

[59] Kosuge T., Yokota M., Sugiyama K., Yamamoto T., Mure T., Yamazawa H. (1985). Studies on bioactive substances in crude drugs used for arthritic diseases in traditional Chinese medicine. II. Isolation and identification of an anti-inflammatory and analgesic principle from the root of Angelica pubescens MAXIM. *Chemical and Pharmaceutical Bulletin*.

[60] Zhao H.-Y., Fan M.-X., Shi J.-L., Wang A.-Q., Li J.-L. (2010). Isolation and structure identification of chemical constituents from seeds of *Lepidium apetalum*. *Chinese Traditional and Herbal Drugs*.

[61] Xiang W.-J., Ma L., Hu L.-H. (2009). C21 steroidal glycosides from *Cynanchum wilfordii*. *Helvetica Chimica Acta*.

[62] Tao X. F., Xu J. L., Zhang R. S. (2009). Research progress on C21 steroidal glycosides from *Asclepiadaceae*. *Chinese Journal of Ethnomedicine and Ethnopharmacy*.

[63] Calvo M. I. (2006). Anti-inflammatory and analgesic activity of the topical preparation of *Verbena officinalis* L.. *Journal of Ethnopharmacology*.

[64] Zhou B., Meng X. (1992). Pharmacological study on *Veronicastrum sibiricum* (L.) Pennell. *China Journal of Chinese Materia Medica*.

[65] Bishayee A., Sarkar A., Chatterjee M. (1995). Hepatoprotective activity of carrot (*Daucus carota* L.) against carbon tetrachloride intoxication in mouse liver. *Journal of Ethnopharmacology*.

[66] Loizzo M. R., Said A., Tundis R. (2009). Antioxidant and antiproliferative activity of *Diospyros lotus* L. extract and isolated compounds. *Plant Foods for Human Nutrition*.

[67] Hua Y., Wang H.-Q. (2004). Chemical components of *Anaphalis sinica* hance. *Journal of the Chinese Chemical Society*.

[68] Peng T., Xing Y. J., Zhang Q. J. (2011). Chemical constituents in herb of *Ranunculus sceleratus*. *Chinese Journal of Experimental Traditional Medical Formulae*.

[69] Zhou J. Q., Xu A. L., Wang G. F. (2009). Study on the determination methods of chlorogenic acids from *Cephalanoplos segetum*. *Journal of Anhui Agricultural Sciences*.

[70] Juteau F., Masotti V., Bessière J. M., Dherbomez M., Viano J. (2002). Antibacterial and antioxidant activities of Artemisia annua essential oil. *Fitoterapia*.

[71] Lin C.-C., Lin J.-M., Yang J.-J., Chuang S.-C., Ujiie T. (1996). Anti-inflammatory and radical scavenge effects of *Arctium lappa*. *American Journal of Chinese Medicine*.

